# Bioenergetic Controls on Microbial Ecophysiology in Marine Sediments

**DOI:** 10.3389/fmicb.2018.00180

**Published:** 2018-02-13

**Authors:** James A. Bradley, Jan P. Amend, Douglas E. LaRowe

**Affiliations:** ^1^Department of Earth Sciences, University of Southern California, Los Angeles, CA, United States; ^2^Department of Biological Sciences, University of Southern California, Los Angeles, CA, United States

**Keywords:** bioenergetics, numerical modeling, dormancy, maintenance energy, geobiology, life in extreme environments, low energy, endogenous and exogenous metabolism

## Abstract

Marine sediments constitute one of the most energy-limited habitats on Earth, in which microorganisms persist over extraordinarily long timescales with very slow metabolisms. This habitat provides an ideal environment in which to study the energetic limits of life. However, the bioenergetic factors that can determine whether microorganisms will grow, lie dormant, or die, as well as the selective environmental pressures that determine energetic trade-offs between growth and maintenance activities, are not well understood. Numerical models will be pivotal in addressing these knowledge gaps. However, models rarely account for the variable physiological states of microorganisms and their demand for energy. Here, we review established modeling constructs for microbial growth rate, yield, maintenance, and physiological state, and then provide a new model that incorporates all of these factors. We discuss this new model in context with its future application to the marine subsurface. Understanding the factors that regulate cell death, physiological state changes, and the provenance of maintenance energy (i.e., endogenous versus exogenous metabolism), is crucial to the design of this model. Further, measurements of growth rate, growth yield, and basal metabolic activity will enable bioenergetic parameters to be better constrained. Last, biomass and biogeochemical rate measurements will enable model simulations to be validated. The insight provided from the development and application of new microbial modeling tools for marine sediments will undoubtedly advance the understanding of the minimum power required to support life, and the ecophysiological strategies that organisms utilize to cope under extreme energy limitation for extended periods of time.

## Introduction

Marine sediments across the globe host a rich microbial biosphere, whose dynamics are important analogs to oligotrophic and extra-terrestrial environments, and whose activity bears a major control on organic carbon (OC) burial and thus global climate. Microbial cells in oligotrophic marine sediments catabolize 10^4^–10^6^ fold more slowly than model organisms in nutrient-rich media (Hoehler and Jørgensen, 2013), yet despite enduring prolonged starvation, they persist over geological timescales (D'Hondt et al., 2015). The vast majority of marine sediments constitute one of the most energy-limited habitats on Earth (Lever et al., 2015) and provide ideal test-cases to study the extreme limits of life over timescales that challenge fundamental notions of what it means to be alive. By studying life at its limit (i.e., low energy, low nutrients), much can be learned about the fundamental ecophysiology of microorganisms. Since most microorganisms in marine sediments appear to be merely surviving rather than growing, factors such as metabolic state and maintenance energy utilization become increasingly important over the long timescales (i.e., thousands to millions of years) associated with this habitat.

Despite much effort to characterize and understand the ecophysiology associated with microorganisms and microbial communities from the deep subsurface biosphere, it is generally still unknown which factors govern whether microorganisms buried in sediments will grow and produce daughter cells, lie dormant for thousands to millions of years, or die at an extremely slow rate. A more comprehensive understanding of the factors that determine physiological state, as well as energy utilized for growth and maintenance, are crucial in addressing these fundamental questions facing deep biosphere research.

Here, we discuss bioenergetics, dormancy and maintenance energy in the subsurface, and consider how quantitative approaches (i.e., modeling) provide opportunities to complement ongoing research. We provide mathematical constructs for simulating microbial growth, yield, maintenance activities, and physiological state changes (i.e., active and dormant), and discuss them in the context of application to the marine subsurface biosphere. We hope to encourage a better integration of theoretical and experimental approaches to subsurface bioenergetics, which we believe is required to advance deep biosphere investigations beyond what can presently be captured by observations alone.

## Bioenergetics as a driver of microbial dynamics in marine sediments

All organisms require energy to stay alive. That energy is ultimately harvested from the catalysis of redox reactions. Exergonic (energy-yielding) reactions are catalyzed within or nearby living cells at some rate to provide power. This power may ultimately be used to fuel endergonic (energy-requiring) reactions to maintain a cellular steady-state and sometimes (but not always) to grow. The amount of energy available from the catalysis of exergonic redox reactions can be determined by calculating the Gibbs energy of a potential reaction under a given set of geochemical conditions. Gibbs energy calculations demonstrate not only which reactions are thermodynamically favorable and thus constitute conceivable catabolic strategies for microorganisms, but also which environmental variables, including temperature, pressure, pH, salinity, and the concentrations of electron donors and terminal electron acceptors, influence the amount of energy available to microorganisms. In marine sediments, these factors are largely driven by the flux and burial of organic and mineral particles, and living organisms, to the ocean floor. Physical processes such as bioturbation, the diffusion of aqueous species including electron acceptors (e.g., O_2_, ${\text{SO}}_{4}^{2-}$) and secondary redox products (e.g., Fe^2+^, CH_4_, H_2_S), the sorption of OC to mineral surfaces, and mineral precipitation, also alter sediment properties. OC is the primary electron donor for microorganisms in marine sediments (Arndt et al., 2013) and O_2_ and ${\text{SO}}_{4}^{2-}$ are the primary electron acceptors for its oxidation (Thullner et al., 2009).

## Energy for growth and maintenance

Growth yields and cellular maintenance requirements are subject to trade-offs based on selective pressures in different environments (Lele and Watve, 2014). Under low-energy conditions, such as in marine sediments, it is thought that microbial activity is limited, more or less, to maintaining cellular integrity through biomolecular repair and replacement (Westerhoff et al., 1983; Tijhuis et al., 1993; del Giorgio and Cole, 1998; Smith and Prairie, 2004; Carlson et al., 2007; Orcutt et al., 2013). Maintenance activities constitute the sum of activities that do not produce growth (e.g., regeneration of enzymes, maintaining membrane integrity, motility, etc.). Accordingly, maintenance activities potentially constitute a much greater fraction of total power utilized by microbial communities in marine sediments compared to other natural settings, or those grown in laboratories.

However, data from (or representative of) marine sediments are lacking, and an accurate determination of the *in situ* maintenance power utilization of microorganisms in any natural setting is challenging. Empirical approaches are plagued by methodological problems, experimental artifacts, and inconsistencies across studies (Hobson, 1965; Hempfling and Mainzer, 1975; Russell and Baldwin, 1979). Laboratory-determined values of maintenance powers are also likely a gross over-estimation of power requirement in natural settings due to the favorable (high-energy) conditions under which microorganisms are grown in the laboratory compared to the conditions that microorganisms experience in nature (LaRowe and Amend, 2015a). However, by integrating experimental datasets with numerical modeling, LaRowe and Amend (2015b) derived microbial power use by microorganisms from oligotrophic sediments in the South Pacific Gyre that were several orders of magnitude lower than laboratory-measured maintenance powers. Given the extreme energy-limitation of these sediments, the low rates of OC processing, and the net decline in biomass over the multi-million-year timescales over which cells are buried, it can be assumed that cells present in these sediments are not growing, and thus calculated power utilization represents mostly (if not exclusively) maintenance activities.

Maintenance energy for microorganisms in marine sediments might come from (i) endogenous catabolism, i.e., the utilization of biomass (Herbert, 1958), (ii) exogenous catabolism, i.e., the consumption of additional substrate (Pirt, 1965), or (iii) a combination of the two, which might vary depending on thermodynamic and environmental factors (Wang and Post, 2012).

## Activity akin to dormancy

The idealized conditions under which microorganisms are grown in the laboratory rarely occur in nature. The apparent ubiquity of microorganisms in natural environments that exhibit extraordinarily slow growth, intermittent growth, or even no growth, reflects a general lack of available energy (Morita, 1988). Microorganisms in marine sediments may be considered some of the slowest, most energy-limited living organisms on Earth, generally exhibiting levels of activity that are several orders of magnitude lower than anything measured via cultivation (Jørgensen and Boetius, 2007; D'Hondt et al., 2009, 2015; Røy et al., 2012; Hoehler and Jørgensen, 2013; Jørgensen and Marshall, 2016). The typical vegetative-like state of microorganisms in marine sediments is tantamount to dormancy (Jørgensen and Marshall, 2016), a transient and reversible state of low metabolic activity. Dormancy is thought to enable microorganisms to endure extended periods of unfavorable conditions such as energy-limitation, without the need to divide (Lever et al., 2015). Despite the omnipresence of dormant cells in marine sediments, the exact nature of dormancy and of its bioenergetic controls in relation to the marine sedimentary environment are not well understood. For instance, the rate at which energy is used by dormant cells, or the thermodynamic and environmental parameters that initiate or terminate dormancy, are not known. A quantitative approach toward microbial dormancy incorporating bioenergetics is thus required to truly understand the deep biosphere.

## Bioenergetics and modeling

Microbial and geochemical models can help determine the fluxes of energy and material between ecosystem components, disentangle processes that are observed experimentally as a net outcome, and predict the sensitivity and response of ecosystems and geochemical environments to perturbations and changing conditions. Further, they are useful to bridge scales, interpolate between observations, and help identify important data and knowledge gaps. Models are also particularly helpful for deep biosphere investigations since the marine subsurface is notoriously difficult to study using traditional sampling strategies, because of its remoteness and relative inaccessibility, the exceedingly slow rates of energy processing, and the vast timescales over which measurements represent.

A review of the diagenetic models commonly used to simulate the degradation of OC, a process that drives biogeochemical reactions in marine sediments, is presented by Arndt et al. (2013). Thermodynamic models have also been used to quantify the power supply to and demand by microorganisms in marine sediments (e.g., LaRowe and Amend, 2015b), and Gibbs energy calculations have been used to infer what types of reactions microorganisms may be catalyzing in the subsurface (e.g., Teske et al., 2014; McKay et al., 2016; Sylvan et al., 2016). However, the majority of models assume that microbial biomass is in a steady state or has negligible influence beyond transient timescales (Thullner et al., 2005). Models need to be complex enough to describe the required properties and processes of the system, but structurally and mechanistically simple enough to be able to constrain and validate parameters and simulations from available data and literature. At present, a suitable microbial-biogeochemical model for the marine subsurface, capturing the ecophysiological factors discussed here, does not exist. In the following sections, we provide formulations for how such factors may be represented. The models presented here are based on differential equations that describe ecological processes in a mathematical sense. For a comprehensive guide to formulating ecological models, we direct the reader to Soetaert and Herman (2009).

### Heterotrophic growth and organic carbon degradation

The following model (Equations 1 and 2), and some variations of these expressions including logistic growth, a rate limiting term, and mortality, form the basis of many ecosystem models (Soetaert and Herman, 2009; Sierra et al., 2015). Here, heterotrophic growth is dependent on the availability of OC as a substrate, and can be described by:

(1) $$\begin{array}{l} \frac{\delta B}{\delta t}=\left(V_{max}\cdot B\cdot \frac{OC}{K_{OC}+OC}\right)-\left(\alpha \cdot B\right) \end{array}$$

where *B* denotes the concentration of microbial carbon, *t* is time, *V*_*max*_ corresponds to maximum microbial growth rate, *OC* denotes OC concentration (the non-living organic component), *K*_*OC*_ is the half-saturation constant for OC, a kinetic parameter that describes the dependency of microbial growth on OC concentration, and α represents the specific death rate.

**Figure 1:**
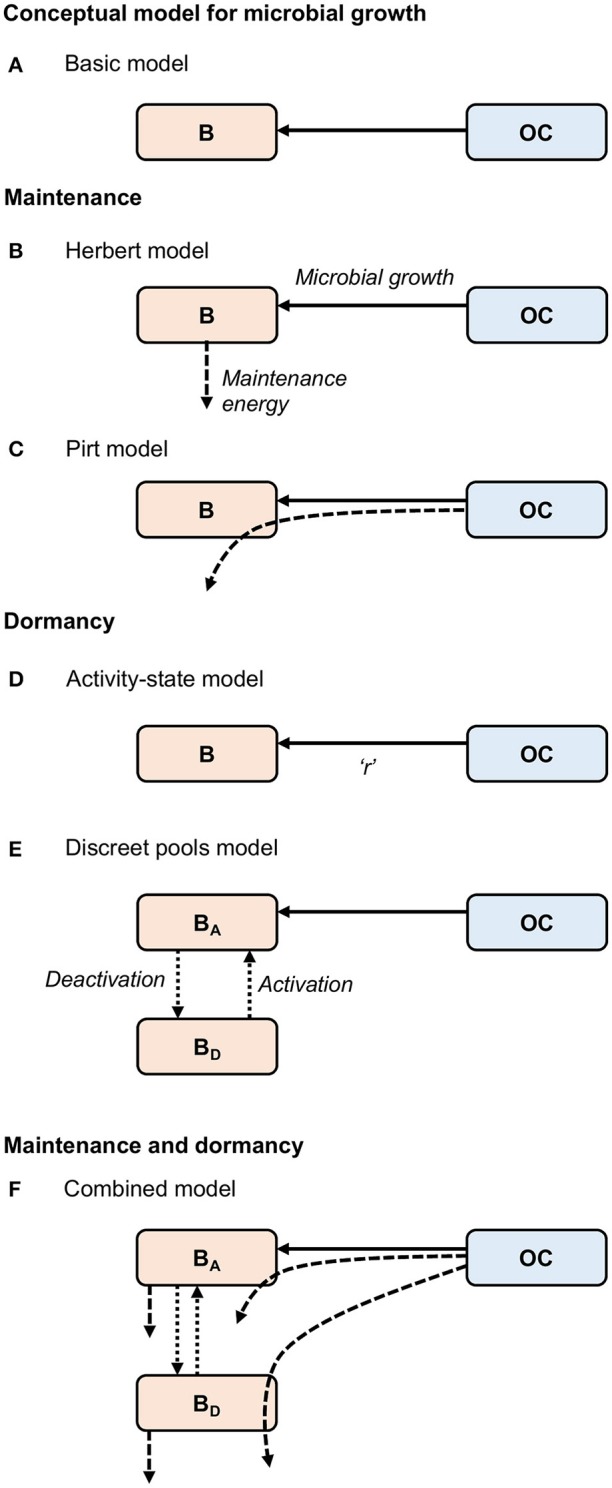
Conceptual models for **(A)** “Basic” microbial growth, maintenance utilizing **(B)** Herbert, and **(C)** Pirt formulations, dormancy utilizing **(D)** “Activity-state” and **(E)** “Discreet pools” approaches, and **(F)** a “Combined model,” developed here, combining formulations for maintenance and dormancy. Solid arrows represent biomass growth, dashed arrows represent maintenance energy, and dotted arrows represent transfers between active (*B*_*A*_) and dormant (*B*_*D*_) states. Microbial death [transfer from biomass (*B*) to organic carbon (*OC*)] is not shown. “*r”* in the “Activity-state” model **(D)** represents the proportion of actively growing microbial biomass (*B*), and varies from zero to one.

The change in OC due to microbial processes can then be represented by:

(2) $$\begin{array}{l} \frac{\delta OC}{\delta t}=-\left(\frac{1}{Y}\cdot V_{max}\cdot B\cdot \frac{OC}{K_{OC}+OC}\right)+\left(\alpha \cdot B\right) \end{array}$$

where *Y* represents an observed growth yield, which is the efficiency of converting carbon into microbial products (Sinsabaugh et al., 2013). The contribution of dead biomass to OC is represented by (α·*B*). Equations (1) and (2) constitute the “Basic” model (Figure 1A). This approach lumps maintenance and growth costs into a single parameter (*Y*), and assumes that all microorganisms are active. Thus, it is not sufficient to describe microbial processes in marine sediments.

### Incorporating maintenance and growth yields

Mechanistically distinguishing between maintenance energy and growth yield is important to accurately quantify bioenergetics in low-energy environments such as marine sediments where non-growing organisms expend a larger proportion of their total power utilization on maintenance.

Two well-known approaches to simulating maintenance energy are provided by Herbert (1958) and Pirt (1965). These approaches differ by the provenance of maintenance energy.

**Figure 2:**
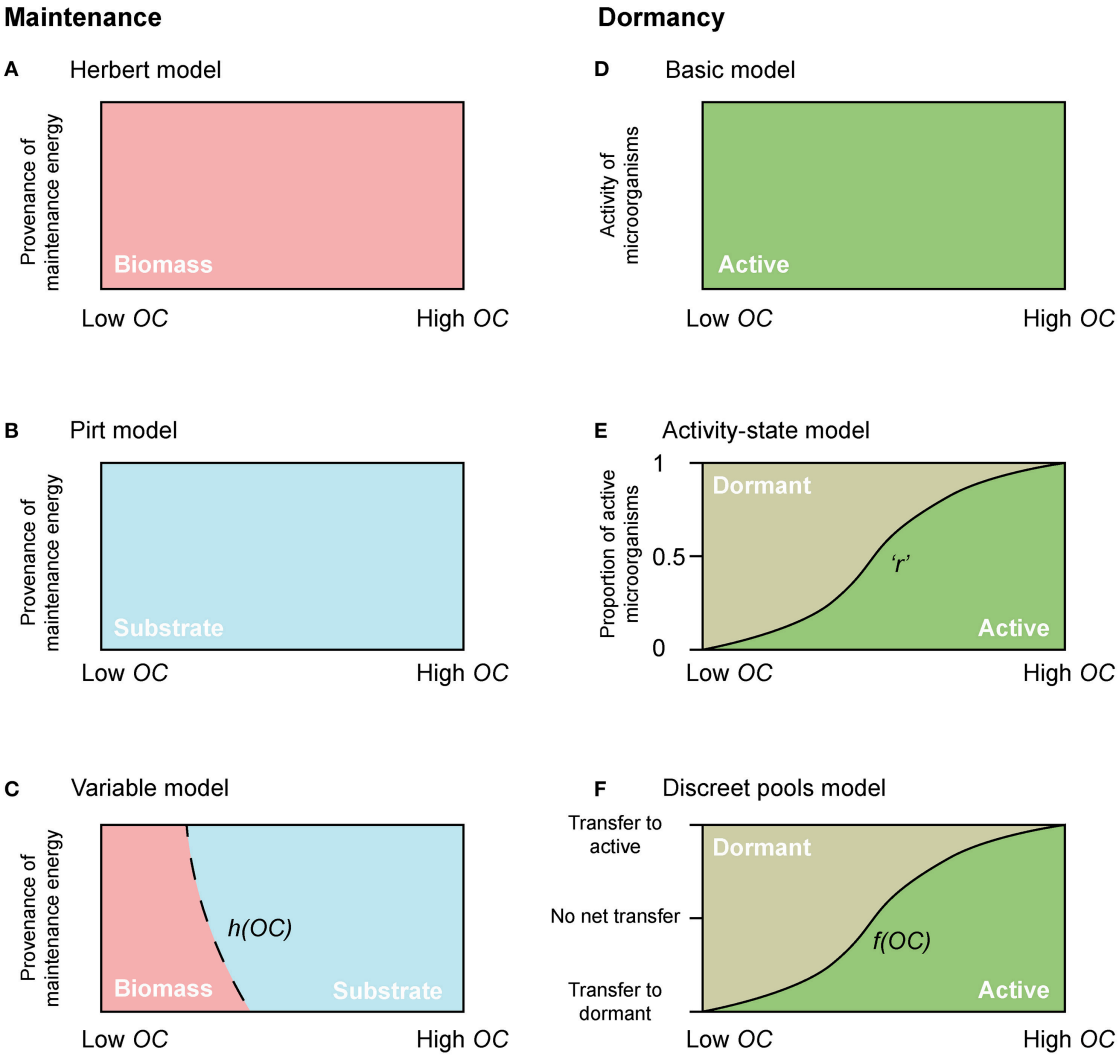
Conceptual diagrams of maintenance energy provenance and dormancy. The dependency of maintenance energy provenance on organic carbon concentration with **(A)** Herbert, **(B)** Pirt, and **(C)** a “Variable” approach. Dependency of physiological state change of microorganisms with **(D)** the “Basic model,” **(E)** the “Activity-state model,” and **(F)** the “Discreet pools model”. “*r”* in the “Activity-state model” represents the proportion of actively growing microbial biomass (*B*), and varies from 0 to 1. “*h(OC)*” in the “Variable model” and “*f(OC)*” in the “Discreet pools model” are functions that vary from 0 to 1.

The first of these model types, commonly referred to as the Herbert approach, considers maintenance costs as endogenous catabolism, i.e., the consumption of biomass (Figures 1B, 2A) (Herbert, 1958; Knapp et al., 1983; Kim and Or, 2016). Thus, the specific maintenance rate is regarded as a negative growth rate:

(3) $$\begin{array}{l} \frac{\delta B}{\delta t}=\left(V_{max}\cdot B\cdot \frac{OC}{K_{OC}+OC}\right)-\left(\alpha \cdot B\right)-\left(a\cdot B\right) \end{array}$$

where *a* indicates maintenance requirement. Correspondingly, the change in OC is:

(4) $$\begin{array}{l} \frac{\delta OC}{\delta t}=-\left(\frac{1}{Y_{G}}\cdot V_{max}\cdot B\cdot \frac{OC}{K_{OC}+OC}\right)+\;\left(\alpha \cdot B\right) \end{array}$$

Here, *Y*_*G*_ represents the “true growth yield” (Pirt, 1965) reflected by the expenditure of energy solely to generate new biomass (Lipson, 2015). This formulation allows for maintenance activities to continue independently of substrate availability, and thus is potentially useful and appropriate for simulating microbial maintenance under substrate-starved conditions, such as in the vast majority of marine sediments. However, this expression does not allow for microorganisms to cover maintenance requirements from substrate, which may occur when substrate is plentiful (Dawes and Ribbons, 1964), and features a maximum specific growth rate (*V*_*max*_) and a “true growth yield” (Y_G_) that are less suitable from an empirical point of view as they cannot be observed or measured directly (Beeftink et al., 1990).

The second model type, commonly referred to as the Pirt approach (Pirt, 1965; Darrah, 1991) considers the additional consumption of substrate for maintenance (Figures 1C, 2B), coupling “Basic” microbial growth (Equation 1) with the consumption of substrate according to:

(5) $$\begin{array}{l} \frac{\delta B}{\delta t}=\left(V_{max}\cdot B\cdot \frac{OC}{K_{OC}+OC}\right)-\left(\alpha \cdot B\right)\\ \frac{\delta OC}{\delta t}=-\left(\frac{1}{Y_{G}}\cdot V_{max}\cdot B\cdot \frac{OC}{K_{OC}+OC}\right)-\left(a\cdot B\right)+\;\left(\alpha \cdot B\right) \end{array}$$

Here, however, the consumption of substrate is numerically possible even in its absence, due to the term that is included for maintenance (*a*·*B*). Thus, this expression could be problematic for simulating marine sediments, because continual uptake of OC in low-energy, OC-poor sediments that characterize the deep biosphere may cause a mass imbalance.

In natural settings, the specific maintenance rate, as well the provenance of maintenance energy, can vary under different environmental conditions (Van Bodegom, 2007). A “Variable” model that allows for environmental factors to dictate the supply of maintenance energy from biomass and/or substrate, adapted from Wang and Post (2012), is:

(6) $$\begin{array}{l} \frac{\delta B}{\delta t}=\left(V_{max}\cdot B\cdot h\left(OC\right)\right)-\left(m_{q}\cdot B\cdot \left(1-h\left(OC\right)\right)\right)-\left(\alpha \cdot B\right) \end{array}$$

where *m*_*q*_ represents the specific maintenance rate, and *h(OC)* is a function that varies from 0 to 1, and allows for microorganisms to cover their maintenance requirements from OC when it is plentiful [*h(OC)* → 1 when *OC* ≫ *K*_*OC*_], but from biomass when OC becomes scarce (*h(0)* = 0) (Figure 2C).

The change in OC is calculated according to:

(7) $$\begin{array}{l} \frac{\delta OC}{\delta t}=-\left(\frac{1}{Y_{G}}\cdot V_{max}\cdot B\cdot h\left(OC\right)\right)-\left(m_{q}\cdot B\cdot h\left(OC\right)\cdot \frac{1}{Y_{G}}\right)\\ +\left(\alpha \cdot B\;\right) \end{array}$$

This model follows the assumptions of (*i*) net negative growth at limiting concentrations of OC (*OC* → 0), (*ii*) no OC consumption when OC is exhausted (*OC* = 0), and (*iii*) no biomass degradation (due to endogenous maintenance) and maximum microbial growth when OC is plentiful (*OC* ≫ *K*_*OC*_).

### Incorporating active and dormant microorganisms

With few exceptions, microbial models do not account for active and dormant biomass, and cells are considered either alive and active, or dead (Figure 2D). Of those models that incorporate multiple physiological states, there are two general approaches. First is to directly regard the active fraction of biomass (i.e., the ratio of active biomass to total living biomass) as a state variable (Figures 1D, 2E) (e.g., Panikov, 1995; Blagodatsky and Richter, 1998; Ingwersen et al., 2008):

(8) $$\begin{array}{l} \frac{\delta B}{\delta t}=r\left(V_{max}\cdot B\cdot \frac{OC}{K_{OC}+OC}-\alpha \cdot B\right) \end{array}$$

where *r* represents the proportion of actively growing biomass (*B*) (0 ≤ *r* ≤ 1), and its value may depend on multiple environmental and biological factors.

The corresponding change in OC is:

(9) $$\begin{array}{l} \frac{\delta OC}{\delta t}=-\;r\left(\frac{1}{Y}\cdot V_{max}\cdot B\cdot \frac{OC}{K_{OC}+OC}\right)+r\left(\alpha \cdot B\right) \end{array}$$

Equations (8) and (9) constitute the “Activity-state” model.

Second is to explicitly separate the total live biomass into active (*B*_*A*_) and dormant (*B*_*D*_) pools (Figures 1E, 2F, e.g., Bär et al., 2002; Stolpovsky et al., 2011):

(10) $$\begin{array}{l} \frac{\delta B_{A}}{\delta t}=V_{max}\cdot B_{A}\cdot \frac{OC}{K_{OC}+OC}-\alpha_{A}\cdot B_{A}-\xi +\epsilon \end{array}$$

(11) $$\begin{array}{l} \frac{\delta B_{D}}{\delta t}=-\alpha_{D}\cdot B_{D}-\epsilon +\xi \end{array}$$

(12) $$\begin{array}{l} \frac{\delta OC}{\delta t}=-\left(\frac{1}{Y_{G}}\cdot V_{max}\cdot B_{A}\cdot \frac{OC}{K_{OC}+OC}\right)+\left(\alpha_{A}\cdot B_{A}\right)\\ +\;\left(\alpha_{D}\cdot B_{D}\right) \end{array}$$

where α_*A*_ and α_*D*_ denote the specific death rate of active and dormant microorganisms respectively, ϵ denotes the transfer of biomass from dormant to active (*B*_*D*_ to *B*_*A*_) (i.e., activation), and ξ denotes the transfer of biomass from active to dormant (*B*_*A*_ to *B*_*D*_) (i.e., deactivation). Equations (10–12) constitute the “Discreet pools” model. The transitions between active and dormant microbial pools are typically dependent on environmental or thermodynamic factors. Under favorable conditions there is net activation, and vice-versa.

### Integrating dormancy and maintenance

We have developed a new approach that allows for biomass and substrate to supply maintenance energy and that resolves the fraction of active and dormant microorganisms, combining the “Variable” model to represent maintenance (Equations 6 and 7) and the “Discreet pools” model to represent dormancy (Equations 10–12):

(13) $$\begin{array}{l} \frac{\delta B_{A}}{\delta t}=\left(V_{max}\cdot B_{A}\cdot h\left(OC\right)\right)-\;\left(m_{q,BA}\cdot B_{A}\cdot \left(1-h\left(OC\right)\right)\right)\\ -\left(\alpha_{A}\cdot B_{A}\right)-\xi +\epsilon \end{array}$$

(14) $$\begin{array}{l} \frac{\delta B_{D}}{\delta t}=-\left(m_{q,BD}\cdot B_{D}\cdot \left(1-h\left(OC\right)\right)\right)-\left(\alpha_{D}\cdot B_{D}\right)-\epsilon +\xi \end{array}$$

(15) $$\begin{array}{l} \frac{\delta OC}{\delta t}=-\left(\frac{1}{Y_{G}}\cdot V_{max}\cdot B_{A}\cdot h\left(OC\right)\right)-\left(m_{q,BA}\cdot B_{A}\cdot h\left(OC\right)\cdot \frac{1}{Y_{G}}\right)\\ -\left(m_{q,BD}\cdot B_{D}\cdot h\left(OC\right)\cdot \frac{1}{Y_{G}}\right)+\left(\alpha_{A}\cdot B_{A}\right)+\;\left(\alpha_{D}\cdot B_{D}\right) \end{array}$$

where *m*_*qBA*_ and *m*_*qBD*_ denote specific maintenance requirements of active and dormant microorganisms respectively (Figure 1F). Dormant cells must carry out maintenance (Johnson et al., 2007), and like living cells, are able to utilize biomass and substrate to cover their maintenance demands. Equations (13–15) constitute the “Combined” model. We believe that this model incorporates sufficiently detailed microbial ecophysiology to form the basis of an ecosystem model for the deep biosphere without being overly complex.

### Integration with experimental data

We are confident that the “Combined” numerical approach outlined here can be used as a foundation to effectively simulate microbial processes in marine sediments across a range of scales. Plausible values for parameters may be taken from existing datasets and modeling studies (e.g., Stolpovsky et al., 2011; Lomstein et al., 2012; Arndt et al., 2013). We also stress that this approach would be improved by future laboratory and field investigations considering the following measurements:

- Microbial growth rate (*V*_*max*_).
- True growth yield, *Y*_*G*_.
- Baseline rates of cellular metabolic activity, equivalent to the specific maintenance requirements of active and dormant microorganisms (*m*_*q,BA*_ and *m*_*q,BD*_).
- The environmental conditions or biological factors under which maintenance energy is supplied by either substrate or biomass (*h(OC)*).
- The environmental conditions or biological factors under which microorganisms are active or dormant (ϵ and ξ).
- The causes and rate of cell death for active and dormant microorganisms (α_*A*_ and α_*D*_).

### Model developments and data integration

The “Combined” model provided in Equations (13–15) can be expanded in the following ways:

- Geochemistry: Primary and secondary redox reactions and equilibrium reactions involving important electron donors and acceptors are implemented in existing reaction-transport models, e.g., BRNS (Jourabchi et al., 2005; Thullner et al., 2009).
- Multiple functional groups: Microorganisms can be distinguished and classified based on functional traits (e.g., spore forming, motile) and metabolism (e.g., heterotroph, chemoautotroph).
- Additional biological dependencies: Microorganisms in a natural setting may be limited by the availability of electron donors, electron acceptors, and/or other environmental/geochemical factors. Biological responses to environmental conditions can be accounted for, e.g., via Michaelis-Menten kinetics (Michaelis and Menten, 1913).
- Thermodynamic factors: Explicit determination of thermodynamic factors, such as threshold energy requirements and cell growth yields on an electron-equivalent basis, and the energetic cost of biomass synthesis (Lever et al., 2015; LaRowe and Amend, 2016).
- Implementation in a 1D framework: In order to simulate diagenetic processes over the timescales of burial in a sediment column, transport processes such as advection, diffusion and bioturbation can be implemented in a 1D framework (Jourabchi et al., 2005; Arndt et al., 2013).

## Outlook

The insight provided from the development and application of a new microbial modeling tool for the deep marine subsurface will undoubtedly advance the current understanding of the minimum energy requirements to support life in marine sediments, and the ecophysiological strategies that organisms utilize to survive low-energy conditions. Such insight might then explain the extraordinary persistence of microbial communities that endure unfavorable conditions over geological timescales.

## Author contributions

All authors listed have made a substantial, direct and intellectual contribution to the work, and approved it for publication.

### Conflict of interest statement

The authors declare that the research was conducted in the absence of any commercial or financial relationships that could be construed as a potential conflict of interest.
